# Supplementary material for: The mechanical impact of col11a2 loss on joints; col11a2 mutant zebrafish show changes to joint development and function, which leads to early-onset osteoarthritis
Source: Philos Trans R Soc Lond B Biol Sci. 2018 Sep 24;373(1759):20170335. doi: 10.1098/rstb.2017.0335 (PMC6158203; doi:10.1098/rstb.2017.0335)
Supplement: Supplementary figures [file rstb20170335supp1.pdf]

The mechanical impact of *col11a2* loss on joints; *col11a2* mutant zebrafish show changes to joint development and function, which leads to early onset osteoarthritis.

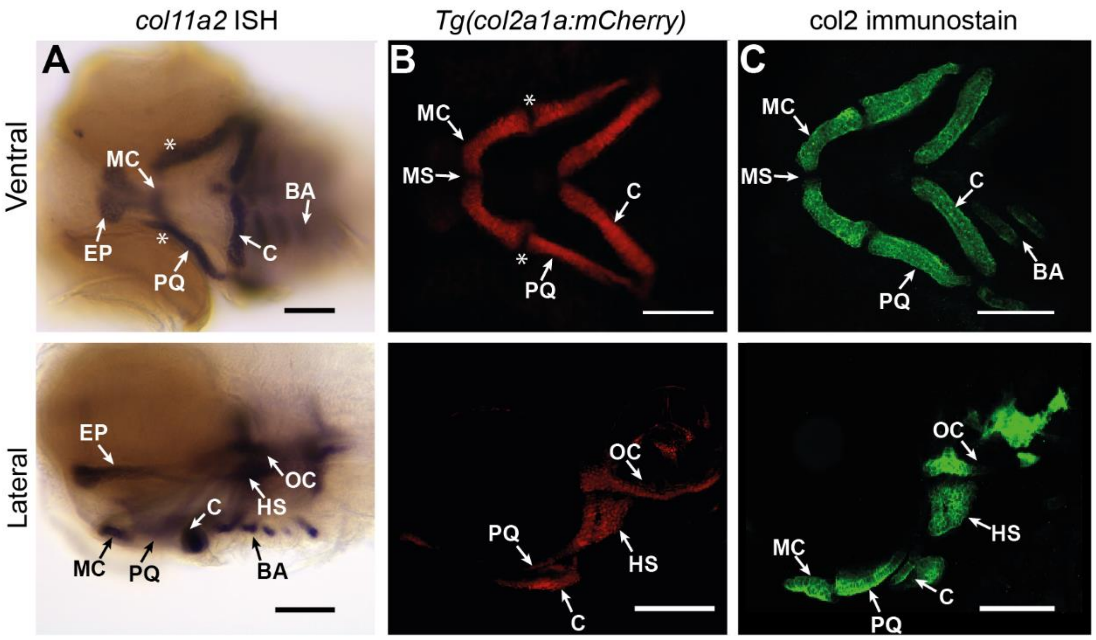

Supplementary Figure 1: *col11a2* and *col2a1* are largely co-expressed in the zebrafish lower jaw. A-C) Ventral and lateral views of zebrafish at 3dpf. A) *In situ* hybridisation (ISH) for *col11a2* shows its expression in the zebrafish Meckel's cartilage (MC), palatoquadrate (PQ), ceratohyal (C), and branchial arches (BA) observed ventrally, and the ethmoid plate (EP), otic capsule (OC) and hyosymplectic (HS) laterally. B) The pattern of expression of *col11a2* overlaps that of *col2a1*, visualised with zebrafish carrying *Tg(col2a1a:mCherry)*. White asterisks = joint region. C) Immunostaining for Type II collagen (col2) indicates protein deposition in the same domains that *col11a2* is expressed, except the jaw joint. Scale bar = 100µm.

Elizabeth A Lawrence*, Erika Kague*, Jessye A Aggleton, Robert L Harniman, Karen A Roddy and Chrissy L Hammond

The mechanical impact of *col11a2* loss on joints; *col11a2* mutant zebrafish show changes to joint development and function, which leads to early onset osteoarthritis.

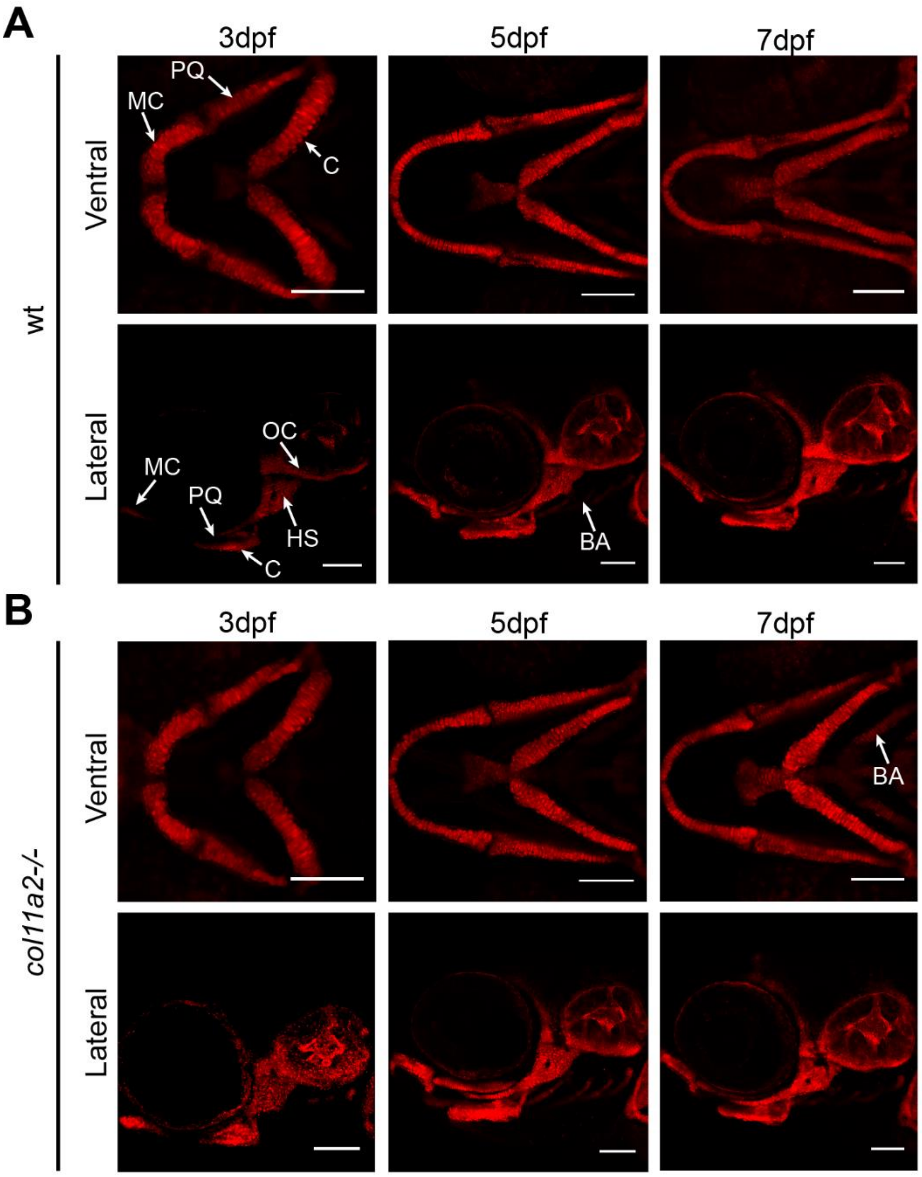

Supplementary Figure 2: *col2a1* expression in craniofacial cartilages is unaffected by loss of *col11a2*. Ventral and lateral views of wt (A) and *col11a2*-/- (B) zebrafish carrying *Tg(col2a1a:mCherry)*. MC = Meckel's cartilage, PQ = palatoquadrate, C = ceratohyal, HS = hyosymplectic, OC = otic capsule and BA = branchial arches, Scale bar = 100µm.

Elizabeth A Lawrence*, Erika Kague*, Jessye A Aggleton, Robert L Harniman, Karen A Roddy and Chrissy L Hammond

The mechanical impact of *col11a2* loss on joints; *col11a2* mutant zebrafish show changes to joint development and function, which leads to early onset osteoarthritis.

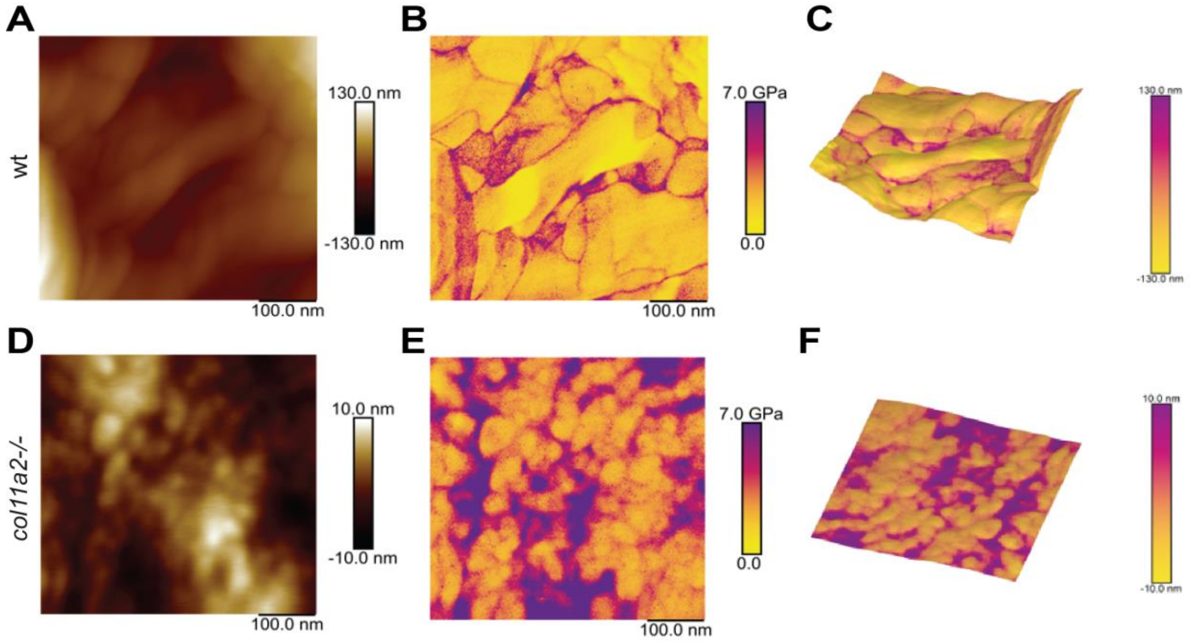

Supplementary Figure 3: Atomic Force Microscopy concurrent measurement of topography and Young's Modulus. (A-C) wt, (D-F) *col11a2-/-* . A marked difference in topography is noted between wt (A) and col11a2-/- (D), with the mutant displaying lower height variation across a given region and comprising of smaller ultra-structure. The difference in ultra-structure is emphasised in the corresponding Young's modulus measurements where larger domains are observed in the wt (B) than the *col11a2-/-* (E), where the smaller domains of the mutant also display a higher Young's modulus. For clarity (C) and (F) display the 3 dimensional surface of the wt and *col11a2-/-* respectively with the corresponding YM overlaid as a colour-map.

Elizabeth A Lawrence*, Erika Kague*, Jessye A Aggleton, Robert L Harniman, Karen A Roddy and Chrissy L Hammond

The mechanical impact of *col11a2* loss on joints; *col11a2* mutant zebrafish show changes to joint development and function, which leads to early onset osteoarthritis.

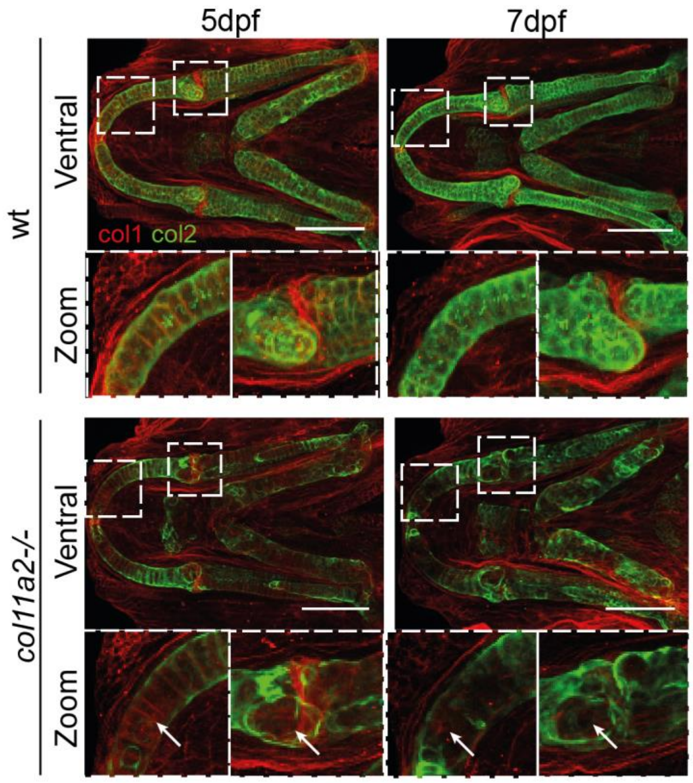

Supplementary Figure 4: Type I collagen is not increased in *col11a2* mutants. Immunostaining for Type I (red) and Type II (green) collagen in wt and *col11a2-/-* at 5 and 7dpf. Dashed insets show areas of higher magnification. White arrows indicate areas of Type II collagen loss, where Type I collagen is unchanged. Scale bar = 100µm.

Elizabeth A Lawrence*, Erika Kague*, Jessye A Aggleton, Robert L Harniman, Karen A Roddy and Chrissy L Hammond

The mechanical impact of *col11a2* loss on joints; *col11a2* mutant zebrafish show changes to joint development and function, which leads to early onset osteoarthritis.

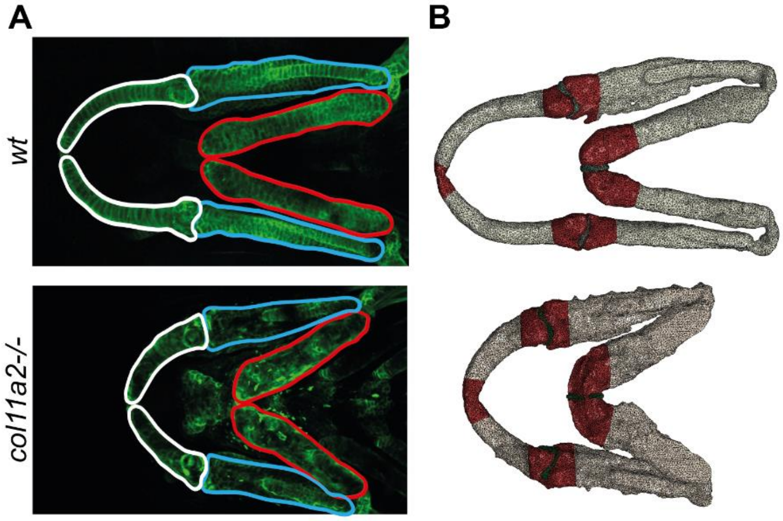

**Table 1**	**Values**	
	**Wild type**	**Mutant**
**Hypertrophic chondrocytes**	7.55 MPa	30.53 MPa
**Immature chondrocytes**	4.29 MPa	5.48 MPa
**Joint**	0.25 MPa	0.25 MPa
**Poissons ratio**	0.25	0.25

Supplementary Figure 5: Segmentation of lower jaw elements and assignment of material property values. A) The Meckel's cartilage (white), palatoquadrate (blue) and ceratohyal (red) were segmented from confocal images of 7dpf wt and *col11a2-/-* zebrafish. B) Material properties from Atomic Force Microscopy were applied to the corresponding regions of the lower jaw. Dark red = immature chondrocytes in the joint regions, grey = hypertrophic chondrocytes towards the middle of the cartilage element, and green = joint. Table 1) Material property values used for Finite Element model generation (MPa = Megapascals).

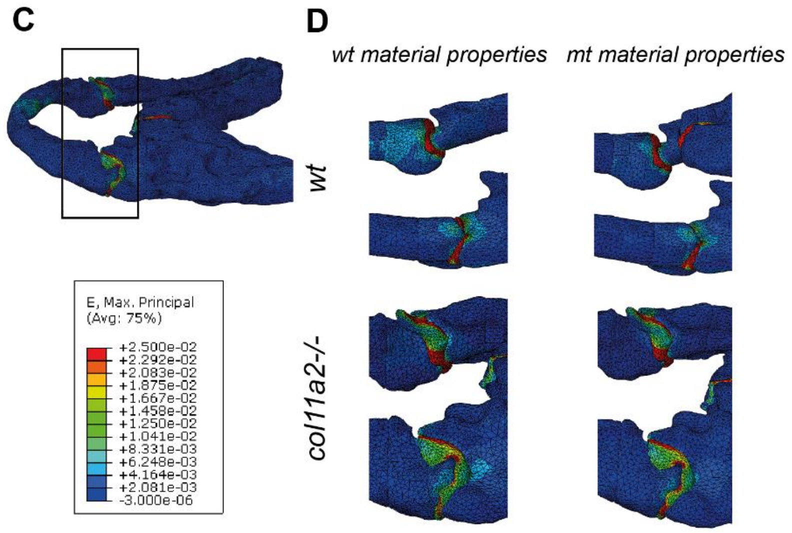

**Table 2**	**Average joint values**	
	**wt mat.**	**mt mat.**
wt	4.41E-02	5.14E-02
col11a2-/-	1.51E-02	1.63E-02

Joints in the opening stage displaying maximum principal strains. C) Region shown in the close-up images and the scale used. D) Interzone joints (between the Meckel's cartilage and palatoquadrate) from each model were probed for maximum principal strain values. Table 2) the average values from probing the joints in each model (mat. = material properties).

Elizabeth A Lawrence*, Erika Kague*, Jessye A Aggleton, Robert L Harniman, Karen A Roddy and Chrissy L Hammond

The mechanical impact of *col11a2* loss on joints; *col11a2* mutant zebrafish show changes to joint development and function, which leads to early onset osteoarthritis.

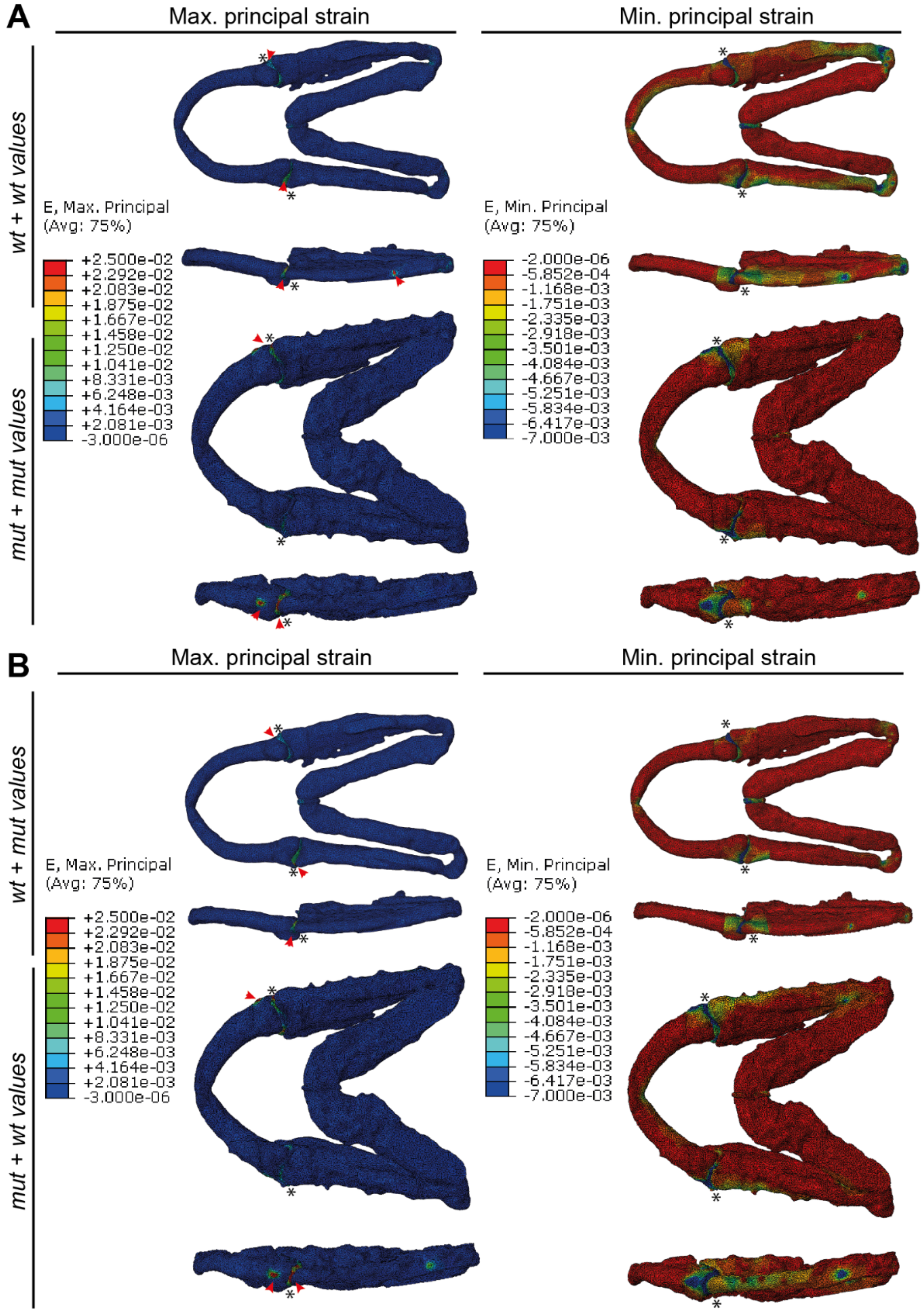

Supplementary Figure 6: Shape changes in *col11a2* zebrafish mutants have a greater affect on jaw biomechanics than material property changes. A, B) Finite Element (FE) models of maximum (E. Max) and minimum (E. Min) principal strain during mouth closing in 7dpf wt and *col11a2-/-* zebrafish. Red arrowheads = areas of high strain, black asterisks = jaw joint. A) wt jaw shape with wt material properties and *col11a2-/-* shape with *col11a2-/-* material properties. B) wt shape with *col11a2-/-* material properties and *col11a2-/-* shape with wt material properties. Ventral and lateral views shown for each.

Elizabeth A Lawrence*, Erika Kague*, Jessye A Aggleton, Robert L Harniman, Karen A Roddy and Chrissy L Hammond

The mechanical impact of *col11a2* loss on joints; *col11a2* mutant zebrafish show changes to joint development and function, which leads to early onset osteoarthritis.

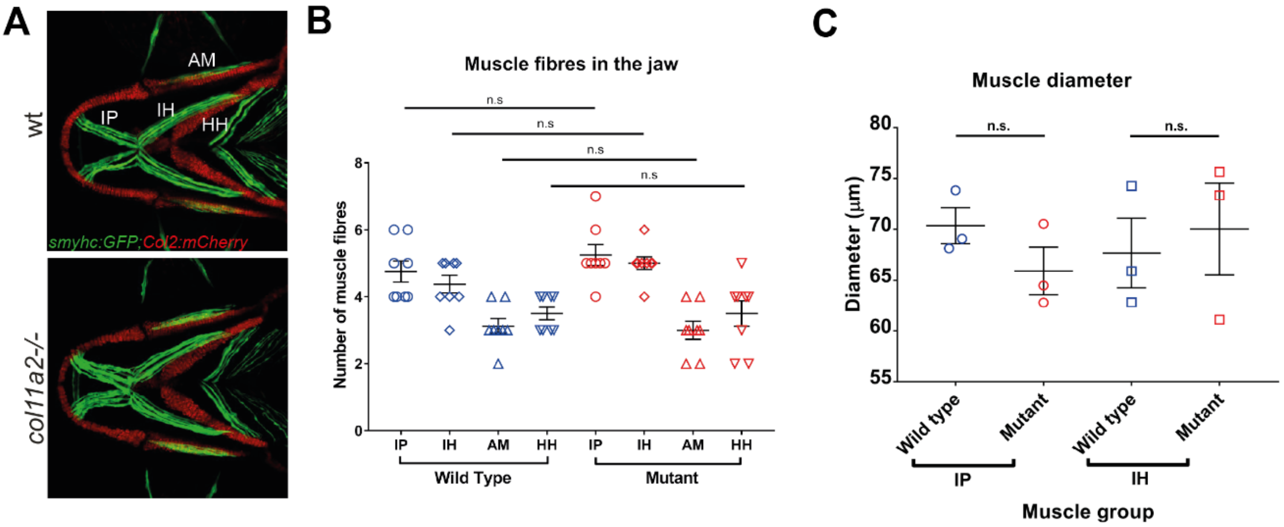

Supplementary Figure 7: Mutation of *col11a2* does not alter muscle fibre number or diameter at 5dpf. A) *smyhc:GFP;col2a1:mCherry* transgenic line shows slow myosin in muscle (green) and Type II collagen (red) in wt and *col11a2-/-* zebrafish at 5dpf. B) Quantification of muscle fibre number in four muscles in the jaw in wt and *col11a2-/-* zebrafish (n = 5 and 8 respectively). C) Quantification of muscle diameter in two muscles in the lower jaw, observed through birefringence in wt and *col11a2-/-* zebrafish at 5dpf (n = 3 for all). Locations of muscles are labelled and shown in (A) (IP = Intermandibularis posterior, IH = Interhyoideus, AM = Adductor mandibulae, HH = hyohyoideus). Student's unpaired t-tests were performed in B and C, data is mean with SEM. Scale bar = 100µm. n.s = P>0.05

Elizabeth A Lawrence*, Erika Kague*, Jessye A Aggleton, Robert L Harniman, Karen A Roddy and Chrissy L Hammond
